# Dimerization of a cell-penetrating peptide leads to enhanced cellular uptake and drug delivery

**DOI:** 10.3762/bjoc.8.204

**Published:** 2012-10-18

**Authors:** Jan Hoyer, Ulrich Schatzschneider, Michaela Schulz-Siegmund, Ines Neundorf

**Affiliations:** 1 Institute of Biochemistry, Faculty of Biosciences, Pharmacy and Psychology, University of Leipzig, Brüderstraße 34, D-04103 Leipzig, Germany; 2 Institute for Biochemistry, Department of Chemistry, University of Cologne, Zülpicher Straße 47, D-50674 Cologne, Germany; 3 Institute of Inorganic Chemistry, Julius-Maximilians-Universität Würzburg, Am Hubland, D-97074 Würzburg, Germany; 4 Institute of Pharmaceutical Technology, Faculty of Biosciences, Pharmacy and Psychology, University of Leipzig, Eilenburger Straße 15A, D-04317 Leipzig, Germany, University of Leipzig

**Keywords:** anti-tumor agents, cell-penetrating peptides, drug delivery, internalization studies, organometallic complexes, peptides

## Abstract

Over the past 20 years, cell-penetrating peptides (CPPs) have gained tremendous interest due to their ability to deliver a variety of therapeutically active molecules that would otherwise be unable to cross the cellular membrane due to their size or hydrophilicity. Recently, we reported on the identification of a novel CPP, sC18, which is derived from the C-terminus of the 18 kDa cationic antimicrobial protein. Furthermore, we demonstrated successful application of sC18 for the delivery of functionalized cyclopentadienyl manganese tricarbonyl (cymantrene) complexes to tumor cell lines, inducing high cellular toxicity. In order to increase the potential of the organometallic complexes to kill tumor cells, we were looking for a way to enhance cellular uptake. Therefore, we designed a branched dimeric variant of sC18, (sC18)_2_, which was shown to have a dramatically improved capacity to internalize into various cell lines, even primary cells, using flow cytometry and fluorescence microscopy. Cell viability assays indicated increased cytotoxicity of the dimer presumably caused by membrane leakage; however, this effect turned out to be dependent on the specific cell type. Finally, we could show that conjugation of a functionalized cymantrene with (sC18)_2_ leads to significant reduction of its IC_50_ value in tumor cells compared to the respective sC18 conjugate, proving that dimerization is a useful method to increase the drug-delivery potential of a cell-penetrating peptide.

## Introduction

A substantial problem concerning promising drug candidates is often their incapacity to reach their full therapeutic potential due to limited bioavailability and cellular uptake. In recent years, cell-penetrating peptides (CPPs) emerged as an encouraging tool to overcome this obstacle owing to their ability to autonomously cross the cellular membrane in a receptor-independent manner. This enables them to deliver a large variety of cargo molecules for therapy and diagnosis, as could be successfully shown for, e.g., cytostatics [1], proteins [2], oligonucleotides [3–4] and nanoparticles [5]. A common feature of CPPs is their typically high content in basic arginine and lysine residues, leading to a positive net charge of the peptides, which is considered to be crucial for initial membrane interaction through binding to negatively charged phospholipids and glycosaminoglycans [6]. Endocytic and non-endocytic processes have been proposed to be involved in cellular uptake; however, the exact mechanism triggering internalization is still under debate and was shown to depend on several factors such as structure and concentration of the CPP as well as the cargo to be transported and the specific cell line [7].

Recently, we identified a novel cell-penetrating peptide, sC18 [8], derived from the C-terminal domain of CAP18 (18 kDa antimicrobial protein), which is found in rabbit leukocytes and was shown to bind to negatively charged lipopolysaccharides of Gram-negative bacteria to inhibit their pathogenic activity [9]. We were able to successfully apply sC18 for tumor imaging by conjugation with a metal chelator and a tumor-homing agent, which accumulates in hypoxic tissue [10]. Furthermore, we reported on the delivery of functionalized cyclopentadienyl manganese tricarbonyl (cymantrene) complexes with the help of sC18, which lead to significant induction of cytotoxicity in tumor cells [11–13], which was even more pronounced after introduction of an enzymatic cleavage site [12]. However, the aim of the present study was to investigate whether the cytotoxic effect of these organometallic complexes can be further increased by conjugating them to a dimeric variant of sC18, (sC18)_2_, since previous studies demonstrated improvement of the delivery of various kinds of cargo by CPP oligomerization [14–15].

## Results and Discussion

### Uptake studies

**Figure 1:**
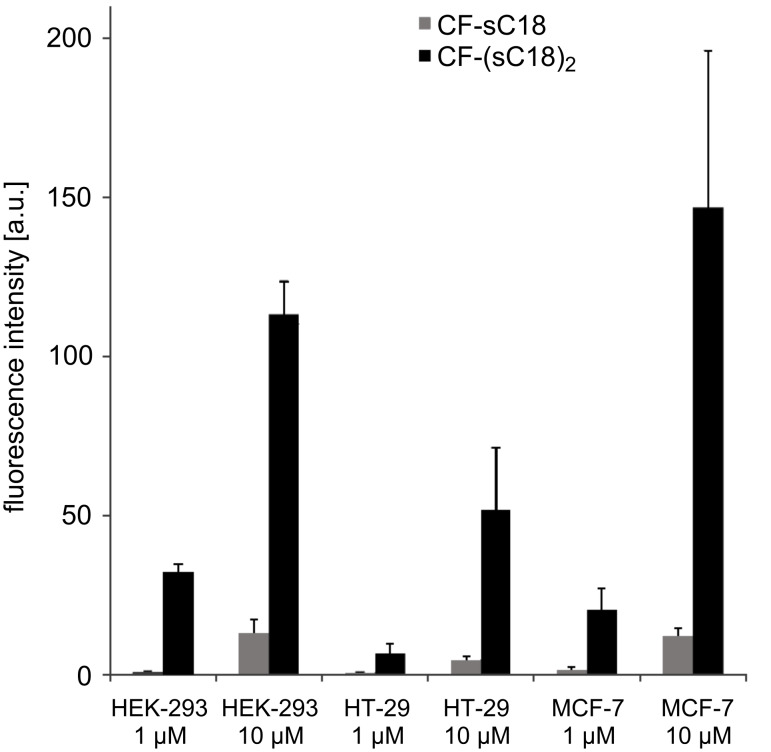
Flow cytometric uptake studies of carboxyfluorescein-labeled (sC18)_2_ in HEK-293 (human embryonic kidney) cells, HT-29 cells (human colon adenocarcinoma) and MCF-7 cells (human breast adenocarcinoma) with sC18 as reference after 1 h incubation. Experiments were conducted in duplicate with *n* = 2. Error bars represent the standard deviation.

We reasoned that dimerization of sC18 would be beneficial for cellular uptake since the local concentration of the monomeric unit at the membrane would be increased. Instead of simply synthesizing a linear peptide with two consecutive sC18 sequences, we opted for a branched version of (sC18)_2_ by introducing the second unit at the side chain of Lys^4^. This ensures that both N-termini of the monomeric units remain free and hence the general structure of the dimer is unaltered upon symmetric introduction of N-terminal modifications. To test the hypothesis of improved internalization behavior of the dimer, we conducted flow-cytometric cellular uptake studies with sC18 versus (sC18)_2_ in various cell lines after N-terminal labeling with carboxyfluorescein (labeling of the dimer occurred at the N-terminus of the first sC18 unit). A dramatic increase of intracellular fluorescence that was 10-fold or higher in all cell lines was observed (Figure 1). Even at a peptide concentration as low as 1 µM significant uptake of (sC18)_2_ after 1 h incubation was achieved, whereas the parent peptide sC18 almost failed to internalize at this concentration. In human embryonic kidney (HEK-293) cells and MCF-7 cells (human breast adenocarcinoma) the uptake rate of (sC18)_2_ at 1 µM was significantly higher even when compared to sC18 at a concentration of 10 µM (p ≤ 0.05). When comparing the different cell lines, the uptake rate of (sC18)_2_ at 1 µM decreases in the order HEK-293 > MCF-7 > HT-29 (p ≤ 0.05). Similarly, at 10 µM, the amount of (sC18)_2_ taken up by HT-29 cells (human colon adenocarcinoma) is still less than half compared to the other cell lines; however, no statistically significant difference between HEK-293 and MCF-7 was observed.

The drastically enhanced uptake of the dimer compared to the monomeric peptide is in contrast to previous studies with the TAT peptide, which is similar to sC18 with respect to the number of arginine and lysine residues and the overall charge of the peptide. Dimerization of TAT turned out to have no or little effect on translocation through cellular [14,16] or model [17] membranes. Only a branched trimeric variant of TAT was observed to have a major impact on the internalization behavior [14].

**Figure 2:**
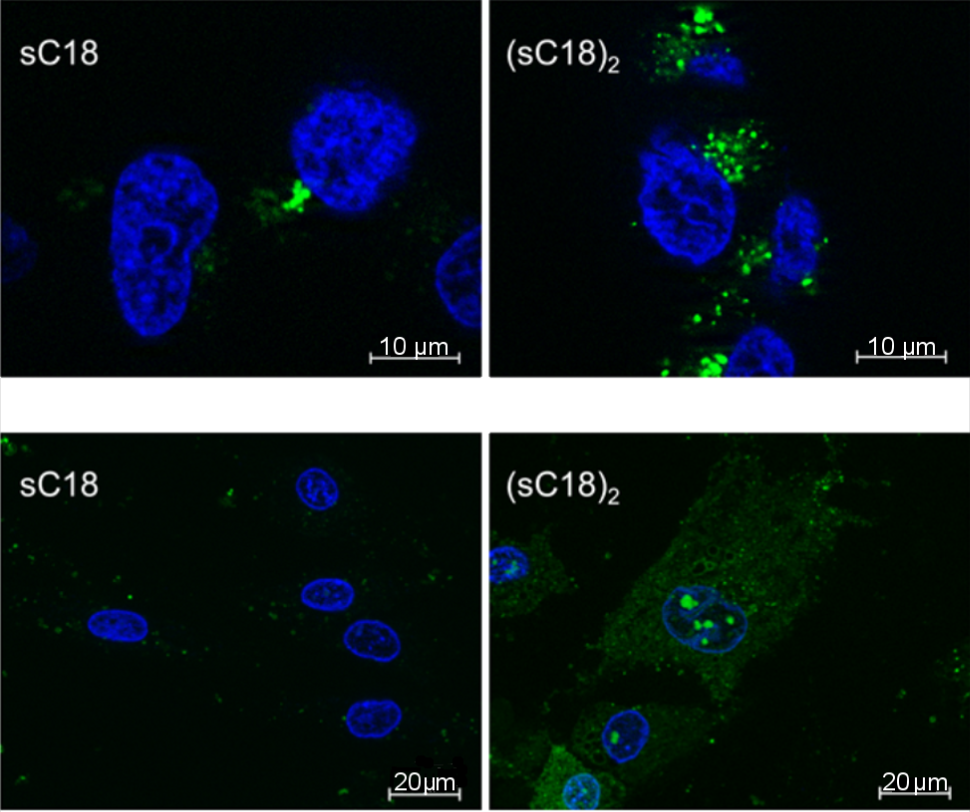
Top: Fluorescence microscopic images of unfixed HEK-293 cells after 30 min incubation with 1 µM CF-sC18 and CF-(sC18)_2_. Bottom: Fluorescence microscopic images of unfixed hADSC after 1 h incubation with 25 µM CF-sC18 and CF-(sC18)_2_. Blue: Hoechst33342 nuclear dye, green: carboxyfluorescein-labeled peptides. Images taken with a 63× oil immersion objective.

In order to gain insight into the mechanism of cell entry of (sC18)_2_, we investigated the intracellular distribution pattern of the fluorescently-labeled peptide in HEK-293 by fluorescence microscopy (Figure 2). The punctate uptake pattern speaks in favor of an endocytic internalization mode and is also observed for sC18, which is in line with previous reports [8]. Therefore, the general uptake mechanism in this cell line seems not to be altered upon dimerization.

With this potent cell-penetrating peptide at hand, we were interested as to whether it is also able to internalize into primary cells, which are much less susceptible to CPP-based intracellular delivery than immortalized or tumor cell lines. We indeed observed bright intracellular fluorescence when incubating human adipose tissue-derived stem cells for 1 h with (sC18)_2_ at 25 µM as opposed to sC18, which shows hardly any detectable uptake (Figure 2). Interestingly, the peptide is evenly distributed throughout the cells, indicating that a large fraction of (sC18)_2_ was able to reach the cytosol. Whether this is due to a different mechanism of uptake or to improved endosomal escape requires further investigation. Importantly, no effect on cell morphology is apparent and, thus, no cytotoxicity at a concentration that is sufficient for very efficient peptide internalization.

### Cytotoxicity of (sC18)_2_

**Figure 3:**
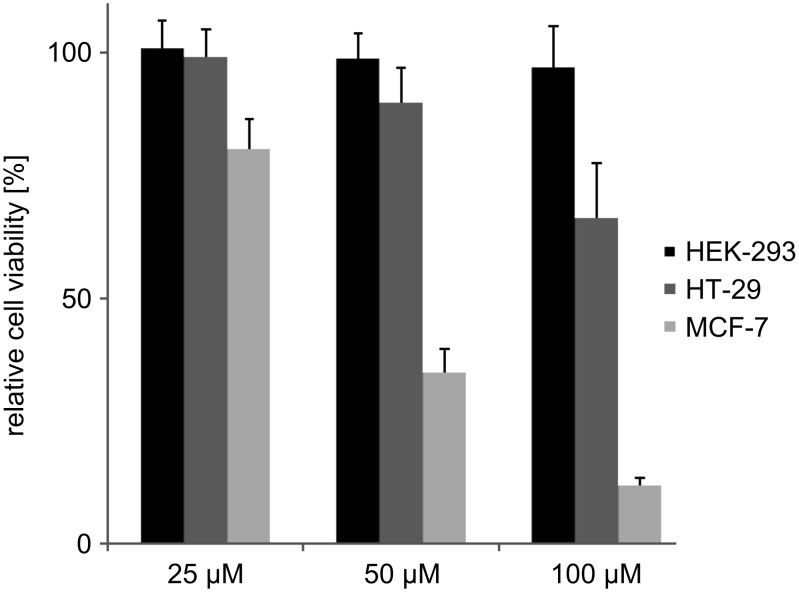
Cell viability of different cell lines after 24 h incubation with (sC18)_2_ at different concentrations as determined by a resazurin-based cell viability assay. Experiments were conducted in triplicate with *n* = 2. Error bars represent the standard deviation.

The effect of the dimeric sC18 on the survival of HEK-293, HT-29 and MCF-7 cells was determined by means of a resazurin-based cell viability assay. After 24 h incubation, a cell-type-dependent cytotoxicity profile of (sC18)_2_ was observed (Figure 3). While HEK-293 cells remained unharmed even at high peptide concentrations up to 100 µM, a steady decrease of cell viability was induced in the tumor cell lines, which was particularly obvious in MCF-7 cells. At least for HEK-293 and MCF-7, this effect seemed to be in no way related to different intracellular amounts of peptide since both cell lines showed equal propensity to take up (sC18)_2_ as was shown above. The significant induction of cytotoxicity in MCF-7 and HT-29 is in plain contrast to the monomeric peptide, which did not cause any loss of cell viability in previous studies [8].

**Figure 4:**
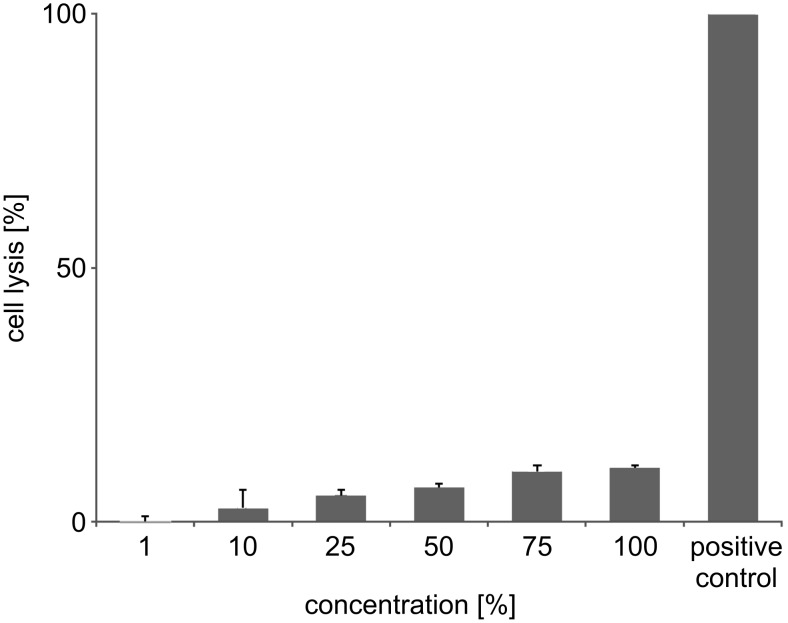
Cell lysis of HEK-293 cells induced by (sC18)_2_ after 1 h incubation. Experiments were conducted in triplicate with *n* = 2. Error bars represent the standard deviation.

Since (sC18)_2_ possesses a net charge of +17 and due to the fact that it has been shown for oligoarginines that the higher the number of positive charges, the higher is the propensity of the peptide to induce pore formation in the cellular membrane [18], we performed membrane integrity assays measuring the amount of lactate dehydrogenase (LDH) released from the cells when the membrane becomes prone to leaking. In the case of MCF-7, high levels of extracellular LDH even after 1 h incubation with 30 µM (sC18)_2_ attested to a high extent of membrane destabilization, which eventually leads to cell death (Figure 8), suggesting membrane disruption as the cause for the cellular toxicity of the dimeric peptide owing to its high number of basic residues. Extracellular LDH levels in HT-29 were much smaller, which is in line with the results of the resazurin-based cell viability assay, as well as the observation that HEK-293 cells only show a little membrane leakage even at elevated (sC18)_2_ concentrations (Figure 4). It can thus be hypothesized that the cytotoxic behavior of the peptide is related to the individual membrane composition of each cell line.

The finding that the dimer exhibits significant cytotoxic effects while the monomer does not is consistent with previous studies that demonstrated the increase of membrane leakage and cytotoxicity with the number of arginine residues in oligoarginines [19] as well as increased cytotoxicity for oligomeric CPPs in general [20].

### Synthesis and characterization of (sC18)_2_ bioconjugates

**Scheme 1:**
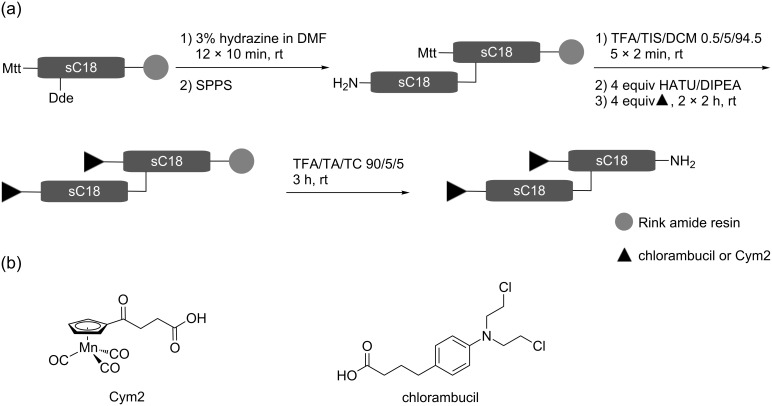
Synthesis of (sC18)_2_ bioconjugates (a) and chemical structures of the coupled anti-tumor agents (b). Mtt: 4-methyltrityl chloride; Dde: 1-(4,4-dimethyl-2,6-dioxocyclohex-1-ylidene)ethyl; SPPS: solid-phase peptide synthesis; TFA: trifluoroacetic acid; TIS: triisopropylsilane; HATU: *O*-(7-azabenzotriazol-1-yl)-1,1,3,3-tetramethyluronium hexafluorophosphate; DIPEA: diisopropylethylamine; TA: thioanisole; TC: *p*-thiocresol.

**Figure 5:**
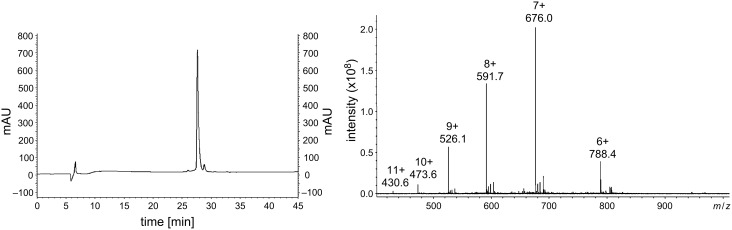
Chromatogram and ESI-MS of purified Cym2-GFL-(sC18)_2_. The gradient was 10→60% acetonitrile in water over 45 min.

In order to evaluate the potential of the sC18 dimer to effectively deliver cytotoxic drugs into cells and even enhance the cytotoxicity of functionalized cymantrenes, the synthesis of which has previously been reported [11], we synthesized a single (**1**) and a double conjugate (**2**) of (sC18)_2_ and Cym2 (Table 1, Scheme 1). For comparison, we also synthesized bioconjugates with known cytostatic agents: the common DNA alkylating anticancer therapeutic chlorambucil (Cbl, **3**), which may induce apoptosis, and the cell impermeable proapoptotic peptide (KLAKLAK)_2_ (PAD, proapoptotic domain peptide) [21] **4**. All conjugates were synthesized with an enzymatic cleavage site for the peptidase cathepsin B (Gly-Phe-Leu-Gly), the expression of which is up-regulated in tumor cells [22–23], since this approach has been shown to improve intracellular release of the cargo [12,24]. Peptides were obtained in high purity as determined by analytical HPLC, and their identity was confirmed by mass spectrometry (Table 2). However, for compound **2** a partial loss of one Mn(CO)_3_ unit of the organometallic complex was observed to some extent, which was considered to be negligible for the biological testing. An exemplary HPLC chromatogram and ESI-MS spectrum is shown in Figure 5. Double conjugates were synthesized by an orthogonal protecting-group strategy involving highly acid-labile 4-methyltrityl (Mtt) protection (Scheme 1), which can be cleaved in the presence of *tert*-butyloxycarbonyl (Boc). Indeed, we did not observe concomitant deprotection of lysine side chains, which was favored by the fact that Mtt groups situated at the N-terminus are even more acid-sensitive than *N*^ε^-Mtt groups [25].

**Table 1:** Peptide sequences.^a^

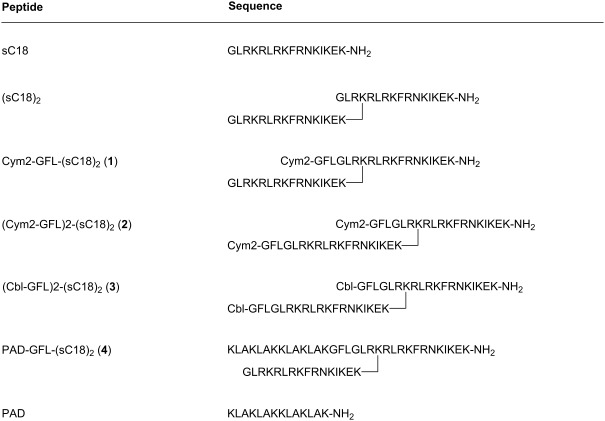

^a^Cbl: chlorambucil.

**Table 2:** Analytical data and IC_50_ values of the (sC18)_2_ bioconjugates.^a^

Peptide	MW_calc_	MW_exp_	Purity [%]	IC_50_ [µM]	
				HT-29	MCF-7

**1**	4722.8	4725.4^b^	>95	33.2 ± 2.2	11.8 ± 0.4
**2**	5326.0	5328.9^b^	>90	21.1 ± 2.0	6.2 ± 0.9
**3**	5324.1	5324.1^c^	>95	26.0 ± 1.2	8.9 ± 0.3
**4**	5941.9	5941.0^c^	>99	14.3 ± 1.4	3.6 ± 1.5

^a^Monoisotopic masses given, purity determined by analytical HPLC; ^b^ESI-MS; ^c^MALDI-MS.

**Figure 6:**
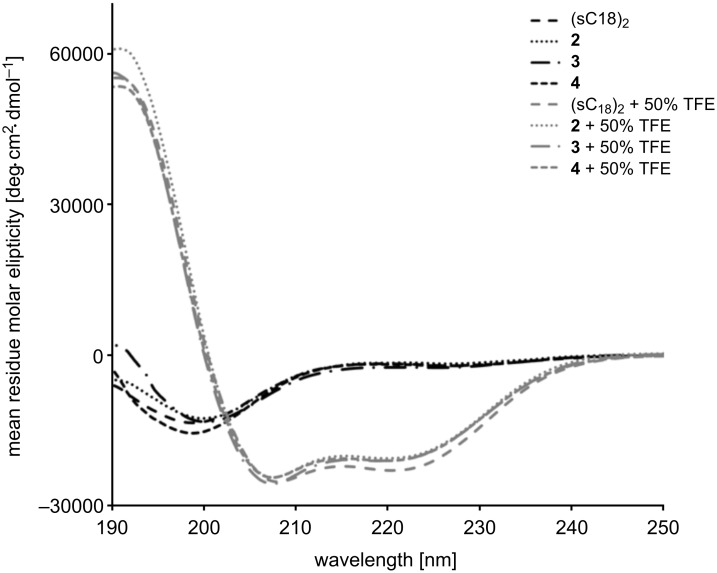
Circular dichroism spectra of the (sC18)_2_ conjugates. Spectra were acquired in 10 mM phosphate buffer with or without 50% trifluoroethanol (TFE), as indicated.

The structural analysis of the (sC18)_2_ conjugates by circular dichroism spectroscopy did not reveal any change of the secondary structure with respect to the parent peptide (Figure 6). They all exhibit a random coil structure in phosphate buffer with transition to an α-helical structure in the helix-inducing environment of trifluoroethanol, which has already been shown for monomeric sC18 [8]. Thus, in this case, no significant influence of the cargo on the uptake of (sC18)_2_ is expected, since this is largely determined by the structure of the CPP.

**Figure 7:**
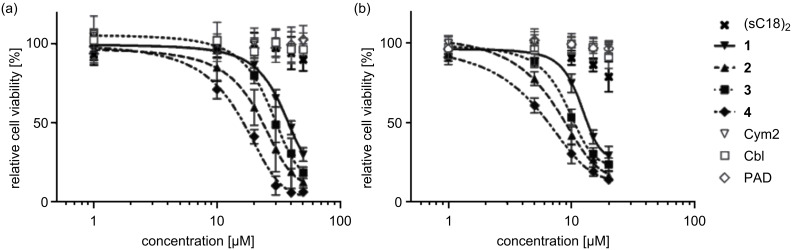
Cell viability of (a) HT-29 and (b) MCF-7 cells after 24 h incubation with the (sC18)_2_ conjugates at different concentrations, as determined by a resazurin-based cell viability assay. Experiments were conducted in triplicate with *n* = 2. Error bars represent standard deviation. Curves obtained by nonlinear regression (sigmoidal dose–response). For IC_50_ values see Table 2.

The bioconjugates were tested for cytotoxicity in MCF-7 and HT-29 in the presence of serum by using a resazurin-based cell viability assay. These are well-established tumor cell lines that allow for direct comparison of the results with previous studies. All substances were shown to exhibit significant cytotoxicity in both cell lines (Figure 7). IC_50_ values are given in Table 2. Importantly, the unconjugated cytostatic compounds did not have any effect on cell viability at any of the concentrations tested, which is confirmed by microscopic images of HT-29 that do not reveal any morphological aberration except in the case of incubation with the bioconjugates. This is in agreement with previous studies for Cym2 [12] and Cbl [26] and is also plausible for PAD, since it was shown to interact with mitochondrial membranes; however, it does not cause cell death unless it is internalized into the cytosol [27]. The failure of the cytostatic compounds to induce cytotoxicity is due to their limited capability to cross the cellular membrane. Likewise, the CPP alone did not affect cell viability of HT-29 cells over the whole concentration range tested, while in MCF-7 the IC_50_ values of **1–4** are still well below the onset of cytotoxic effects, which were only observed to some extent above a concentration of 15 µM. Compound **2** showed significantly increased cytotoxicity compared to **1**, demonstrating the advantage of the double conjugate approach. Importantly, when using the dimer instead of monomeric sC18, which was used in previous studies [12], the IC_50_ values of the functionalized cymantrene could be significantly reduced to 21 µM in HT-29 and 6 µM in MCF-7. Interestingly, Cym2 and Cbl were shown to be similarly effective in both cell lines, whereas the highest cytotoxicity was induced by the PAD conjugate.

**Figure 8:**
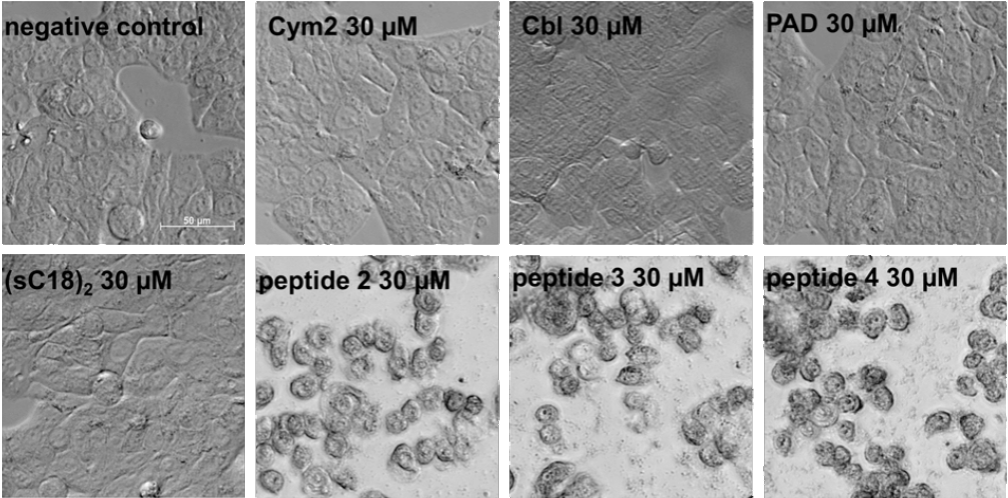
Brightfield microscopic images of unfixed HT-29 cells after 24 h incubation with the (sC18)_2_ conjugates at 30 µM. Scale bar: 50 µm.

**Figure 9:**
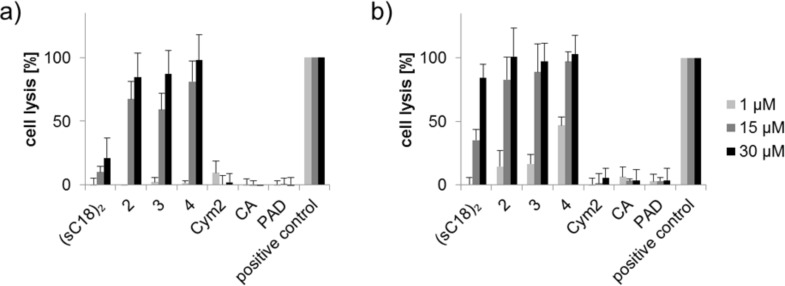
Cell lysis of (a) HT-29 and (b) MCF-7 cells induced by the (sC18)_2_ conjugates at different concentrations after 1 h incubation. Experiments were conducted in triplicate with *n* = 2. Error bars represent the standard deviation.

The mechanism that eventually leads to cell death has not yet been elucidated for the functionalized cymantrenes; however, it was observed that it is mainly induced by necrosis, albeit apoptosis could also be demonstrated to some extent [12]. The high degree of membrane leakage even after 1 h and, thus, the rapidly occurring induction of cell death again speak in favor of mostly necrotic cell death. This is also true for compounds **3** and **4**, which exert the same effect on cell morphology (Figure 8) and induce an equal amount of LDH release (Figure 9). This is surprising at least for compound **4**, since several PAD–CPP conjugates were shown to induce apoptosis upon cytosolic delivery [28–29]. It is possible, though, that the even higher positive net charge (+23) of **4** due to the additional lysine residues of the cargo peptide leads to further membrane destabilization, which eventually becomes irreversible and induces cell death. Thus, (sC18)_2_ may not be suitable for the delivery of positively charged cargo.

In conclusion, we could demonstrate that dimerization of a cell-penetrating peptide, sC18, leads to dramatically enhanced cellular uptake in various cell lines, but also increases cytotoxicity in a cell-type-dependent manner. The dimer also turned out to efficiently internalize into primary cells that are commonly less susceptible to standard delivery methods. Furthermore, we were able to effectively transport cytostatic compounds into tumor cell lines, observing a significant decrease of the IC_50_ values of Cym2 compared to previous studies, further increasing the therapeutic potential of functionalized cymantrenes.

## Experimental

### Materials

*N*^α^-Fmoc-protected amino acids were purchased from IRIS Biotech (Marktredwitz, Germany), the following side-chain protecting groups were chosen: *tert*-butyloxy (*t*-BuO) for Glu; *tert*-butyloxycarbonyl (Boc) and 1-(4,4-dimethyl-2,6-dioxocyclohex-1-ylidene)ethyl (Dde) for Lys. 4-(2’,4’-Dimethoxyphenyl-Fmoc-aminomethyl)phenoxy (Rink amide) resin and 4-methyltrityl chloride were obtained from Novabiochem (Darmstadt, Germany). *O*-(7-Azabenzotriazol-1-yl)-1,1,3,3-tetramethyluronium hexafluorophosphate (HATU), *N*,*N*-diisopropylethylamine (DIEA), thioanisole, *p*-thiocresole, piperidine, trifluoroacetic acid, trypan blue, 5(6)-carboxyfluorescein (CF) and 2,2,2-trifluoroethanol were purchased from Fluka (Taufkirchen, Germany). Chlorambucil (Cbl) and the resazurin-based in vitro toxicology assay kit were purchased from Sigma-Aldrich (Taufkirchen, Germany). *N*,*N-*Dimethylformamide (DMF), dichloromethane, and diethyl ether were obtained from Biosolve (Valkenswaard, Netherlands). Acetonitrile was obtained from Merck (Darmstadt, Germany). The CytoTox-One homogeneous membrane integrity assay was from Promega. For cell culture, the following media and supplements were used: Dulbecco’s modified Eagle’s medium (DMEM), Ham’s F12 (without L-glutamine), OptiMEM, Dulbecco’s phosphate buffered saline (PBS) without calcium and magnesium, fetal bovine serum (FBS), L-glutamine and trypsin/EDTA (all purchased from PAA, Linz, Austria, or Gibco Life Technologies, Karlsruhe, Germany). Cell culture flasks (75 cm^2^) and 96-well plates were from TPP (Trasadingen, Switzerland), 48-well plates were purchased from Greiner Bio-One (Frickenhausen, Germany) and 8-well µ-slides were from ibidi (Martinsried, München Germany). MCF-7, HT-29 and HEK-293 cell lines were kindly provided by Prof. Dr. A. G. Beck-Sickinger.

#### Peptide synthesis

The peptides used were synthesized as described previously [30] by automated solid-phase peptide synthesis (SPPS) on a multiple Syro II peptide synthesizer (MultiSynTech, Witten, Germany) following Fmoc/*t*-Bu-strategy utilizing a double-coupling procedure and in situ activation with Oxyma/DIC. Synthesis of branched (sC18)_2_ was achieved by N-terminal coupling of Dde-Lys(Fmoc)-OH to sC18(5-16) and subsequent automated elongation of the peptide chain at the lysine side chain by using *N*^α^-Boc protected glycine at the terminal position. Cleavage of the Dde group was achieved by treatment with a 3% solution of hydrazine in DMF for 12 × 10 min. Subsequently, the peptide was elongated to its final length by automated SPPS. *N*-terminal coupling of Cym2 or 5(6)-carboxyfluorescein was carried out by using 2 equiv of the substance to be coupled and activation with 2 equiv of HATU/DIPEA in DMF under vigorous shaking for 2 × 2 h. CF-polymers were cleaved by treatment with 20% piperidine for 45 min.

For the synthesis of double conjugates of (sC18)_2_ with Cym2 or Cbl (Scheme 1), the N-terminus of the first sC18 unit was protected by using 10 equiv of Mtt and 1 equiv of DIPEA in DCM under vigorous shaking for 18 h at room temperature. After cleavage of the Dde group at the side chain of Lys^4^ and automated elongation of the peptide chain, the Mtt group was selectively removed by treatment with 0.5% TFA and 5% TIS in DCM for 5 × 2 min. Coupling of the cytostatic compound was carried out by using 4 equiv of either Cym2 or Cbl, and activation with 4 equiv of HATU/DIPEA in DMF under vigorous shaking for 2 × 2 h. All peptides were purified by preparative reversed-phase HPLC using a binary eluent system consisting of 0.1% TFA in water and 0.08% TFA in acetonitrile and analyzed by MALDI or ESIMS, and analytical RP-HPLC. For the peptide sequences see Table 1.

#### Circular dichroism spectroscopy

Circular dichroism spectra were recorded on a Jasco spectropolarimeter J 715 at 20 °C in 10 mM phosphate buffer (pH 7.0) with or without addition of 50% trifluoroethanol, respectively. The concentration of the peptides was 20 µM.

#### Cell culture

HEK-293 cells (human embryonic kidney epithelium transformed with adenovirus 5 DNA) were grown to subconfluency in 75 cm^2^ culture flasks at 37 °C and 5% CO_2_ in a humidified atmosphere by using DMEM/Ham’s F12 (without L-glutamine), which contained 15% heat-inactivated FBS. MCF-7 cells (human breast adenocarcinoma) were grown under the same standard growth conditions in DMEM/Ham’s F12 containing 2 mM L-glutamine and 10% FBS. HT-29 cells (human colon adenocarcinoma) were grown in RPMI 1640 supplemented with 10% FBS. Freshly isolated human-adipose-tissue-derived stem cells (hADSC) were grown in DMEM high glucose (4.5 g/L) containing 1% penicillin/streptomycin and 10% FBS.

#### Internalization studies

For peptide-uptake studies by flow cytometry, cells were seeded in a 48-well plate and grown to 60–70% confluence. After incubation at 37 °C for 1 h with 5(6)-carboxyfluorescein-labeled peptides in OptiMEM, the cells were treated with 150 µM trypan blue for 0.5 min to quench extracellular fluorescence and washed twice with PBS, trypsinized and resuspended in standard cell-culture medium. Analyses were performed on a Partec CyFlow ML flow cytometer. Cellular autofluorescence was subtracted. The experiments were carried out with *n* (number of experiments) = 2 in duplicate.

For fluorescence microscopic uptake studies, cells were seeded in an 8-well ibidi plate and grown to 60–70% confluence. The cells were then incubated with CF-labeled peptides in OptiMEM for 30 min at 37 °C. Ten minutes prior to the end of incubation, the nuclei were stained by addition of Hoechst33342 nuclear dye. Finally, the solution was removed, and the cells were treated with a 150 µM trypan blue solution for 0.5 min. After washing twice with PBS, images were taken by using a Zeiss Observer Z1 fluorescence microscope equipped with an ApoTome unit using a 63× oil immersion objective.

#### Cell viability assay

Cytotoxicity was determined by means of a resazurin-based cell-viability assay. Cells were seeded in a 96-well plate, grown to subconfluency and incubated with the substances at different concentrations for 24 h in the presence of serum under standard growth conditions. For the positive control, cells were treated with 70% ethanol for 10 min. After washing, the cell viability was determined relative to untreated cells by using the In Vitro Toxicology Assay Kit from Sigma-Aldrich according to the manufacturer’s protocol. Measurement was performed fluorimetrically at 595 nm (λ_ex_ = 550 nm) on a Tecan SpectraFluor Plus plate reader. Untreated cells were set to 100%. IC_50_ values were calculated by using nonlinear regression (sigmoidal dose–response) with GraphPad Prism. Experiments were carried out with *n* = 2 in triplicate. Microscopic images were taken with a 20× objective on a Zeiss Observer Z1 microscope.

#### LDH release assay

For the membrane leakage assay utilizing the Promega CytoTox-One kit, cells were seeded in a 96-well plate, grown to subconfluency, and incubated with the substances at different concentrations for 1 h in the presence of serum under standard growth conditions. Afterwards, the assay was conducted according to the manufacturer’s protocol including the provided cell lysis positive control. Measurement was performed fluorimetrically at 595 nm (λ_ex_ = 550 nm) on a Tecan SpectraFluor Plus plate reader. The negative control served as blank value, and data were normalized to the positive control. Experiments were carried out with *n* = 2 in triplicate.

## References

[1] Krauss U, Kratz F, Beck-Sickinger A G (2003). J Mol Recognit.

[2] Looi C Y, Imanishi M, Takaki S, Sato M, Chiba N, Sasahara Y, Futaki S, Tsuchiya S, Kumaki S (2011). PLoS One.

[3] El Andaloussi S, Lehto T, Mäger I, Rosenthal-Aizman K, Oprea I I, Simonson O E, Sork H, Ezzat K, Copolovici D M, Kurrikoff K (2011). Nucleic Acids Res.

[4] Trabulo S, Resina S, Simões S, Lebleu B, Pedroso de Lima M C (2010). J Controlled Release.

[5] Yukawa H, Kagami Y, Watanabe M, Oishi K, Miyamoto Y, Okamoto Y, Tokeshi M, Kaji N, Noguchi H, Ono K (2010). Biomaterials.

[6] Ziegler A (2008). Adv Drug Delivery Rev.

[7] Madani F, Lindberg S, Langel Ü, Futaki S, Gräslund A (2011). J Biophys.

[8] Neundorf I, Rennert R, Hoyer J, Schramm F, Löbner K, Kitanovic I, Wölfl S (2009). Pharmaceuticals.

[9] Larrick J W, Hirata M, Balint R F, Lee J, Zhong J, Wright S C (1995). Infect Immun.

[10] Splith K, Bergmann R, Pietzsch J, Neundorf I (2012). ChemMedChem.

[11] Splith K, Neundorf I, Hu W, Peindy N'Dongo H W, Vasylyeva V, Merz K, Schatzschneider U (2010). Dalton Trans.

[12] Splith K, Hu W, Schatzschneider U, Gust R, Ott I, Onambele L A, Prokop A, Neundorf I (2010). Bioconjugate Chem.

[13] Hu W, Splith K, Neundorf I, Merz K, Schatzschneider U (2012). J Biol Inorg Chem.

[14] Angeles-Boza A M, Erazo-Oliveras A, Lee Y-J, Pellois J-P (2010). Bioconjugate Chem.

[15] Said Hassane F, Ivanova G D, Bolewska-Pedyczak E, Abes R, Arzumanov A A, Gait M J, Lebleu B, Gariépy J (2009). Bioconjugate Chem.

[16] Chugh A, Eudes F (2007). Biochim Biophys Acta, Biomembr.

[17] Zhu W L, Shin S Y (2009). J Pept Sci.

[18] Cahill K (2010). Phys Biol.

[19] Tünnemann G, Ter-Avetisyan G, Martin R M, Stöckl M, Herrmann A, Cardoso M C (2008). J Pept Sci.

[20] Park S-H, Doh J, Park S I, Lim J Y, Kim S M, Youn J-I, Jin H-T, Seo S-H, Song M-Y, Sung S Y (2010). Gene Ther.

[21] Javadpour M M, Juban M M, Lo W-C J, Bishop S M, Alberty J B, Cowell S M, Becker C L, McLaughlin M L (1996). J Med Chem.

[22] Foekens J A, Kos J, Peters H A, Krasovec M, Look M P, Cimerman N, Meijer-van Gelder M E, Henzen-Logmans S C, van Putten W L, Klijn J G (1998). J Clin Oncol.

[23] Ebert M P A, Krüger S, Fogeron M-L, Lamer S, Chen J, Pross M, Schulz H-U, Lage H, Heim S, Roessner A (2005). Proteomics.

[24] Miller K, Erez R, Segal E, Shabat D, Satchi-Fainaro R (2009). Angew Chem, Int Ed.

[25] Aletras A, Barlos K, Gatos D, Koutsogianni S, Mamos P (1995). Int J Pept Protein Res.

[26] Myrberg H, Zhang L, Mäe M, Langel Ü (2008). Bioconjugate Chem.

[27] Takeuchi T, Kosuge M, Tadokoro A, Sugiura Y, Nishi M, Kawata M, Sakai N, Matile S, Futaki S (2006). ACS Chem Biol.

[28] Kwon M-K, Nam J-O, Park R-W, Lee B-H, Park J-Y, Byun Y-R, Kim S-Y, Kwon I-C, Kim I-S (2008). Mol Cancer Ther.

[29] Futaki S, Niwa M, Nakase I, Tadokoro A, Zhang Y, Nagaoka M, Wakako N, Sugiura Y (2004). Bioconjugate Chem.

[30] Rennert R, Wespe C, Beck-Sickinger A G, Neundorf I (2006). Biochim Biophys Acta, Biomembr.

